# Evaluating the student nutrition improvement program: regional disparities and nutritional trends in Yunnan, China (2019–2023)

**DOI:** 10.3389/fpubh.2025.1585735

**Published:** 2025-05-26

**Authors:** Fang Xu, Xin Huang, Long Yu, Yadi Yang, Litao Chang

**Affiliations:** ^1^School of Public Health, Kunming Medical University, Kunming, China; ^2^Yunnan Center for Disease Control and Prevention, Kunming, China; ^3^Wuhua District Center for Disease Control and Prevention, Kunming, China

**Keywords:** nutrition improvement program, rural school-aged children, anthropometric indicators, micronutrient supplementation, nutritional disparities

## Abstract

**Background:**

In order to improve the nutrition and health status of rural school-age children and to reduce malnutrition. This study evaluated the effects of the National Nutrition Improvement Program for Rural Compulsory Education Students (NNIP-RCES) in three counties (Midu, Xuanwei, and Xundian) in Yunnan Province, China, in 2019, 2021, and 2023, and the findings highlight regional differences and future nutrition trends.

**Methods:**

Anthropometric indicators (height and weight) and biochemical markers (hemoglobin, Vitamin A, and Vitamin D) were measured and analyzed using linear mixed-effects models to identify temporal changes, regional differences, and the overall effectiveness of the program.

**Results:**

The program led to significant improvements in height (*β* = 0.42, *p* = 0.013), weight (*β* = 0.66, *p* < 0.001), and Vitamin A levels (*β* = 0.011, *p* < 0.001), demonstrating notable progress in physical growth and Vitamin A supplementation. However, hemoglobin levels declined significantly (*β* = −0.38, *p* = 0.005), particularly in Xundian, highlighting ongoing challenges in anemia prevention. Vitamin D levels remained stable with no significant improvements (*p* = 0.488), possibly influenced by factors such as limited dietary variety and seasonal variations in sun exposure. Additionally, considerable disparities in program outcomes were observed among counties, with Midu generally showing superior improvements compared to Xuanwei and Xundian.

**Conclusion:**

While the NNIP-RCES effectively enhanced physical growth and alleviated Vitamin A deficiency, persistent anemia, unchanged Vitamin D levels, and marked regional disparities underscore critical areas requiring further attention. Future policy refinements should focus on targeted interventions addressing anemia prevention, Vitamin D supplementation, enhanced dietary diversity, and equitable resource allocation across regions. Strengthened monitoring frameworks and comprehensive nutrition education initiatives are also recommended to ensure sustainable improvements and serve as a model for global school-based nutritional interventions.

## 1 Introduction

Malnutrition among school-aged children remains a pressing public health concern, particularly in rural and disadvantaged areas. Nutritional deficiencies during critical growth periods can lead to stunted development, impaired cognitive function, and diminished academic performance, perpetuating cycles of poverty and inequity (1). In response, many countries have implemented school-based nutrition programs to mitigate malnutrition and promote educational equity. These interventions are instrumental in addressing the dual burden of undernutrition and micronutrient deficiencies, with significant long-term benefits for physical and cognitive development (2).

In China, to enhance the nutritional health of students in disadvantaged rural areas and promote educational equity, the “National Nutrition Improvement Program for Rural Compulsory Education Students” (NNIP-RCES) was initiated in November 2011. Initially, this program provided a daily nutritional meal subsidy of 3 RMB per student per school day, benefiting students from 699 counties across 22 provinces, particularly those in areas characterized by concentrated poverty. At the end of November 2014, this subsidy increased to 4 RMB per student per day, and further rose to 5 RMB per student per day by 2021. Currently, the program encompasses 727 nationally designated pilot counties and 1,005 locally designated pilot counties across 28 provinces, covering more than half of China’s county-level administrative regions. It involves over 130,000 rural compulsory education schools, benefiting more than 37 million students, representing approximately 25.4% of all compulsory education students nationwide and about 39.6% of students in rural compulsory education.

NNIP-RCES implementation involved standardized nutritional guidelines developed by China’s National Health Commission and Ministry of Education. These guidelines prescribed specific nutritional standards for school meals, including calorie content, protein, and micronutrient requirements. School meal menus were standardized at the county level based on available local agricultural produce and dietary preferences, with adjustments permitted for seasonal availability. Training sessions for school staff, conducted quarterly, ensured adherence to these nutritional guidelines, food safety standards, and hygienic practices. Additionally, local health and education authorities routinely monitored compliance, performed quality inspections, and offered technical support.

Throughout the implementation of NNIP-RCES, the health administration department are responsible for monitoring and assessing students’ nutritional status and providing dietary guidance. The program clearly defines the specific content and operational requirements for nutrition monitoring and evaluation. Between 2012 and 2019, routine nutritional monitoring was conducted across all 699 national pilot counties, with intensive monitoring carried out in 50 selected counties. In 2021, adjustments were made to streamline the regular monitoring content and expand intensive monitoring efforts, integrating dietary guidance more effectively. The monitoring and dietary assessment cycle was also revised to a biennial schedule (3).

Previous studies have reported studies evaluating the effects of NNIP-RCES implementation in other parts of China: Liang et al. (3) reported improvements in height, weight, and vitamin A levels among rural students, but also highlighted ongoing issues with anemia and vitamin D deficiency (4), which are similar to our findings. In addition, studies have found significant differences in program effectiveness due to differences in local management, dietary habits, and socioeconomic backgrounds (5). However, these early studies focused on short-term effects and lacked comprehensive analysis of temporal trends and regional differences.

This study is an extension of previous studies and provides a detailed longitudinal assessment from 2019 to 2023, covering counties with different socioeconomic conditions in Yunnan Province. The study investigated long-term nutritional trends, regional differences, and the effectiveness of NNIP-RCES in different micronutrient deficiencies, providing more nuanced insights and tailored policy recommendations.

This study aims to assess how effectively the NNIP-RCES has been implemented across three counties in Yunnan, China. Specifically, the objectives include: (1) Examining changes over time in key nutritional and health indicators (height, weight, hemoglobin, Vitamin A, and Vitamin D) among rural school-aged children from 2019 to 2023; (2) Investigating variations between counties to understand regional differences in nutritional outcomes and to evaluate the equitable distribution of program benefits; and (3) Analyzing the overall effectiveness of NNIP-RCES in addressing malnutrition and specific micronutrient deficiencies, and highlighting particular areas where further intervention and enhancements are needed.

The results from this study provide valuable insights into both the successes and challenges of the program, clearly identifying areas that need improvement to ensure lasting and meaningful impacts. By addressing regional disparities and refining strategies for micronutrient supplementation, this study provides actionable recommendations for enhancing NNIP-RCES. These insights are beneficial not only to policymakers and program implementers in China but also offer valuable lessons for global efforts aimed at strengthening school-based nutrition interventions in comparable contexts.

## 2 Materials and methods

### 2.1 Study design and setting

This study utilized a longitudinal design to evaluate the effectiveness of the National Nutrition Improvement Program for Rural Compulsory Education Students (NNIP-RCES) in three rural counties of Yunnan Province, China: Midu, Xuanwei, and Xundian. These counties were selected to represent diverse geographic and socioeconomic conditions within the province. Data were collected over three survey years 2019, 2021, and 2023 to capture temporal trends and assess regional disparities in key nutritional indicators.

### 2.2 Study population

Study participants included school-age children (6 to 15 years) enrolled in rural primary and secondary schools. Stratified random sampling was used to ensure adequate representation between the survey counties and schools. Inclusion criteria for study subjects include: (1) participation in NNIP-RCES for at least 6 months prior to the survey; (2) have complete anthropometric and biochemical data, including height, weight, hemoglobin, vitamin A, and vitamin D; and (3) Written informed consent obtained from a parent or legal guardian. Children diagnosed with serious illnesses not related to nutrition are excluded.

A total of 982 students participated in the survey in 2019, with 1,025 students in 2021 and 1,071 students in 2023 taking a total of 3,078 valid observations. Churn between survey years is primarily due to student transfers, absences from data collection days, or incomplete lab records. The overall sample loss was approximately 9.5%, which was within acceptable limits for community-based longitudinal studies in rural settings.

Sampling error estimates were calculated for key anthropometric indicators. At a 95% confidence level, the margin of error ranged from ±0.94 to ±0.96 cm for height, and ±0.75 to ±0.85 kg for weight, indicating a satisfactory level of statistical precision and adequate power to detect time trends in the primary outcomes.

### 2.3 Data collection

#### 2.3.1 Height and weight measurements

Height and weight were measured by trained health professionals following standardized procedures. Height was recorded to the nearest 0.1 cm using a portable stadiometer, and weight was measured to the nearest 0.1 kg using a calibrated digital scale.

#### 2.3.2 Biochemical analysis

After the time of the physical examination is determined, the school is requested to notify the students in advance to participate in the physical examination and blood draw on an empty stomach in the morning on the day of the physical examination, emphasizing fasting for 10–14 h, skipping breakfast, and drinking a small amount of water. Hemoglobin levels were measured using the HemoCue hemoglobin assay, and vitamin A and serum 25-hydroxyvitamin D levels were quantified using high-performance liquid chromatography–tandem mass spectrometry.

### 2.4 Statistical analysis

Descriptive analysis is used to summarize key anthropometric and nutritional indicators, including height, weight, hemoglobin, vitamin A, and vitamin D levels. The mean and standard deviation (SD) of continuous variables were calculated to represent central trends and variability, while the frequency and proportion of categorical variables were reported. Linear mixed-effects models (LME) were applied to evaluate temporal trends in height, weight, hemoglobin, Vitamin A, and Vitamin D levels. The models included fixed effects for survey year and random effects for counties to account for regional variability. Multivariate ANOVA to assess the combined effect of the survey year on all nutritional indicators, and Tukey’s post-hoc test was performed. Principal component analysis (PCA) was employed to determine patterns in nutrient composition and to assess inter-annual changes in key indicators. All statistical tests were two-sided, and a *p*-value < 0.05 was considered to indicate statistical significance.

## 3 Results

### 3.1 Characteristics and distributions of nutritional indicators

The descriptive statistics for key nutritional and health indicators, including height, weight, hemoglobin, Vitamin A, and Vitamin D, are presented in Table 1. Data were collected across all counties (Midu, Xuanwei, Xundian) and three observation years (2019, 2021, and 2023). The results indicate that from 2019 to 2023, average height and weight increased steadily, but this pattern was not observed in all counties. For example, in Xuanwei and Midu, the average weight fluctuates slightly and does not continue to rise from year to year. These inter-county differences highlight regional heterogeneity in program effectiveness, which may be due to differences in local implementation, socioeconomic conditions, or dietary habits.

**Table 1:** Descriptive nutritional indicators by year and county.

Country	Year	Count	Age (Y)	Height (cm)	Weight (kg)	Hemoglobin (g/L)	Vitamin A (mg/mL)	Vitamin D (ng/mL)
Xuanwei	2019	332	11.39 ± 2.48	143.70 ± 14.56	37.55 ± 12.29	145.86 ± 11.54	0.37 ± 0.08	18.04 ± 5.04
	2021	344	10.86 ± 2.64	142.15 ± 16.05	36.87 ± 13.55	142.73 ± 10.07	0.36 ± 0.08	17.66 ± 5.18
	2023	359	10.78 ± 2.64	142.44 ± 16.08	37.94 ± 13.72	145.09 ± 10.07	0.42 ± 0.10	20.01 ± 5.18
Xundian	2019	335	11.14 ± 2.45	136.44 ± 14.86	32.87 ± 11.39	143.20 ± 11.84	0.38 ± 0.08	19.54 ± 4.74
	2021	334	10.66 ± 2.52	140.71 ± 14.91	36.22 ± 12.87	144.75 ± 15.16	0.38 ± 0.09	19.93 ± 5.63
	2023	347	10.74 ± 2.58	143.22 ± 15.26	37.55 ± 12.84	138.58 ± 12.21	0.40 ± 0.08	19.36 ± 5.48
Midu	2019	315	11.71 ± 2.50	145.98 ± 13.73	37.47 ± 11.70	141.90 ± 11.94	0.39 ± 0.09	22.04 ± 4.93
	2021	347	11.27 ± 2.83	144.64 ± 15.87	38.20 ± 12.92	147.48 ± 14.17	0.37 ± 0.09	19.01 ± 5.41
	2023	365	11.19 ± 2.89	145.49 ± 16.68	40.33 ± 15.70	142.90 ± 11.64	0.45 ± 0.10	19.65 ± 5.05

The average height increased from 136.4 ± 14.8 cm in 2019 to 145.9 ± 13.7 cm in 2023, while the average weight increased from 32.9 ± 11.4 kg to 37.6 ± 13.7 kg in the same period. Notably, hemoglobin and vitamin D levels exhibited large county-to-county variability, suggesting regional differences in addressing anemia and micronutrient deficiencies. In contrast, vitamin A levels showed sustained improvement, especially in 2023, which may be related to targeted nutritional interventions.

### 3.2 Temporal trends of anthropometric and nutritional indicators

Linear mixed-effects models (LME) were applied to assess the temporal trends of key anthropometric and nutritional indicators, accounting for county-level variability. The results demonstrated significant positive trends for height (*β* = 0.42, *p* = 0.013) and weight (*β* = 0.66, *p* < 0.001) over the three-year period. Vitamin A levels also exhibited a significant upward trend (*β* = 0.011, *p* < 0.001) (Table 2).

**Table 2:** Temporal trends of anthropometric and nutritional indicators.

Variable	Coefficient (*β*)	Standard error	*p*-value	Interpretation
Height	0.42	0.17	0.013	Significant increase over time
Weight	0.66	0.15	<0.001	Significant increase over time
Hemoglobin	−0.38	0.14	0.005	Slight but significant decrease
Vitamin A	0.011	0.001	<0.001	Significant increase over time
Vitamin D	−0.04	0.058	0.488	No significant trend

In contrast, Vitamin D levels showed no significant temporal trend (*β* = −0.04, *p* = 0.488). Additionally, hemoglobin levels displayed a slight but statistically significant decline over time (*β* = −0.38, *p* = 0.005), indicating potential gaps in anemia-related interventions.

**Figure 1:**
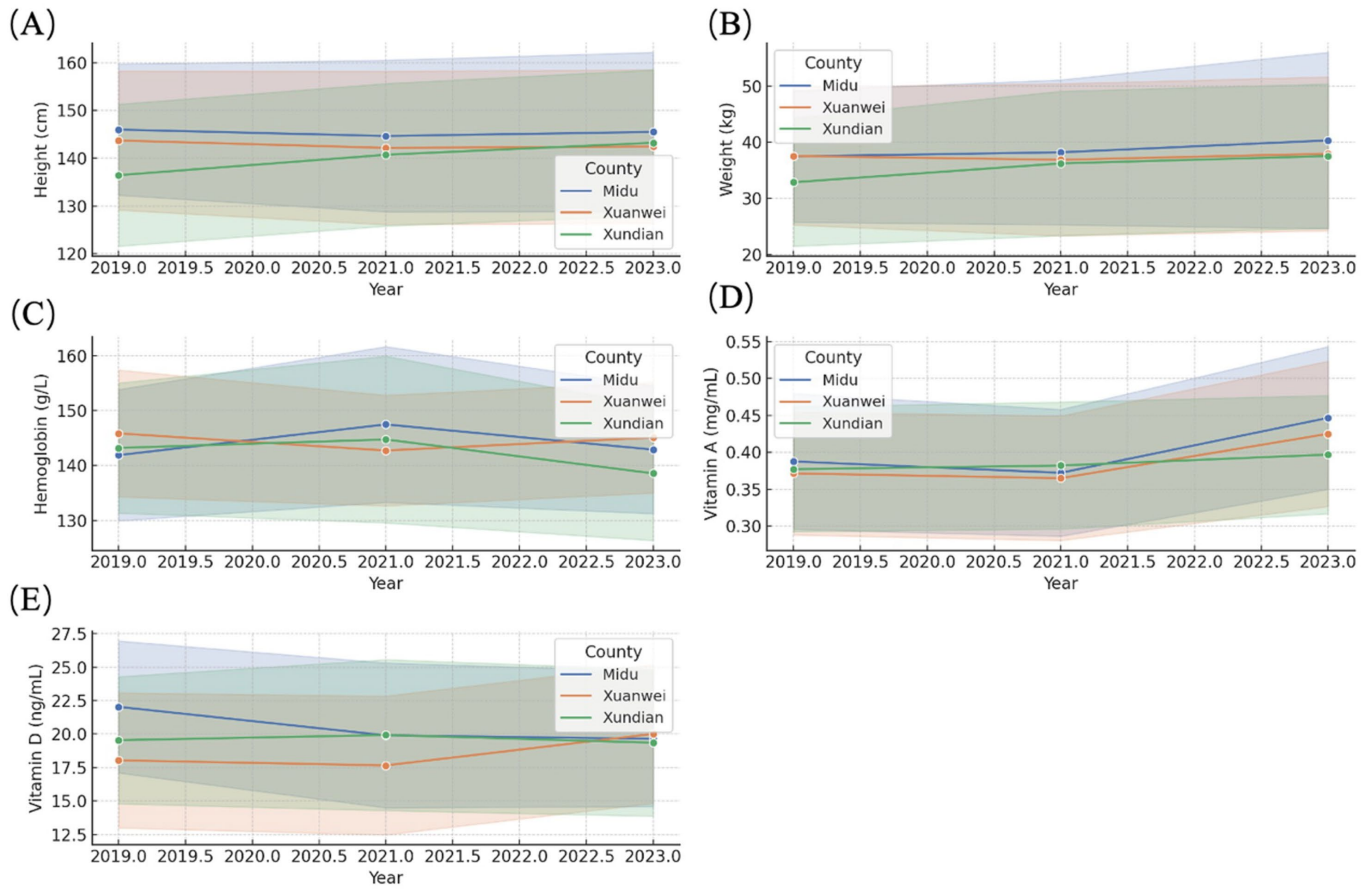
Temporal trends in nutritional indicators among rural school-aged children by county (2019–2023). This figure presents the annual changes in five key nutritional indicators—height **(A)**, weight **(B)**, hemoglobin concentration **(C)**, Vitamin A **(D)**, and Vitamin D **(E)**—measured across three counties (Midu, Xuanwei, and Xundian) participating in the NNIP-RCES. Solid lines represent mean values, and shaded areas indicate standard deviation bands. Overall trends show improvements in physical growth (height and weight) and Vitamin A levels over time, whereas hemoglobin declined slightly, and Vitamin D levels remained relatively stable, with variations observed between counties.

Figure 1 illustrates the temporal trends for these indicators, showing consistent improvements in height, weight, and Vitamin A levels across counties, while Vitamin D and hemoglobin trends were less pronounced. The temporal variations among counties (Midu, Xuanwei, and Xundian) suggest regional differences in the program’s impact, highlighting areas requiring targeted intervention.

The panels in Figure 1 depict the temporal trends for height, weight, hemoglobin, Vitamin A, and Vitamin D levels in Midu, Xuanwei, and Xundian counties. Key observations include: Height and Weight: A consistent upward trend was observed, reflecting the program’s success in promoting growth. Vitamin A: Improved significantly, particularly in Midu, indicating effective micronutrient supplementation. Hemoglobin: Slight declines across counties suggest potential challenges in addressing anemia. Vitamin D: No significant temporal changes, with stable levels across counties.

### 3.3 Differences in nutritional indicators across years

#### 3.3.1 Multivariate analysis of variance

To evaluate whether significant differences existed across years for the key nutritional indicators, a multivariate analysis of variance (MANOVA) was conducted. The pairwise correlations among the variables are shown in Table 3. Results indicate that height and weight were highly correlated (r = 0.867), reflecting their strong association in representing physical growth. Moderate correlations were observed between height and hemoglobin (r = 0.430) as well as between weight and hemoglobin (r = 0.400), suggesting the interplay between growth and oxygen transport capacity. Notably, Vitamin A exhibited a weak positive correlation with height (r = 0.262) and weight (r = 0.313), while Vitamin D showed a weak negative correlation with height (r = −0.281).

**Table 3:** Correlations among nutritional indicators (*r*).

Variable	Height	Weight	Hemoglobin	Vitamin A	Vitamin D
Height	1.000	0.867	0.430	0.262	−0.281
Weight	0.867	1.000	0.400	0.313	−0.253
Hemoglobin	0.430	0.400	1.000	0.225	−0.064
Vitamin A	0.262	0.313	0.225	1.000	0.047
Vitamin D	−0.281	−0.253	−0.064	0.047	1.000

MANOVA results demonstrated that the combined effects of year and nutritional indicators were statistically significant. Specifically, Wilks’ Lambda (0.944, *p* < 0.05) indicated significant differences in the multivariate profile of nutritional indicators across years (Table 4). Further analysis using Pillai’s Trace, Roy’s Largest Root, and Hotelling-Lawley Trace supported these findings, confirming the robustness of the statistical results.

**Table 4:** MANOVA results of nutritional indicators by year.

Effect	Statistic	Value	*F* value	*p* value
Intercept	Wilks’ Lambda	0.944	36.667	*p* < 0.05
	Pillai’s Trace	0.056	36.667	
	Hotelling-Lawley Trace	0.060	36.667	
	Roy’s Largest Root	0.060	36.667	
Year	Wilks’ Lambda	0.945	35.742	
	Pillai’s Trace	0.055	35.742	
	Hotelling-Lawley Trace	0.058	35.742	
	Roy’s Largest Root	0.058	35.742	

#### 3.3.2 Distributions of nutritional indicators by year

**Figure 2:**
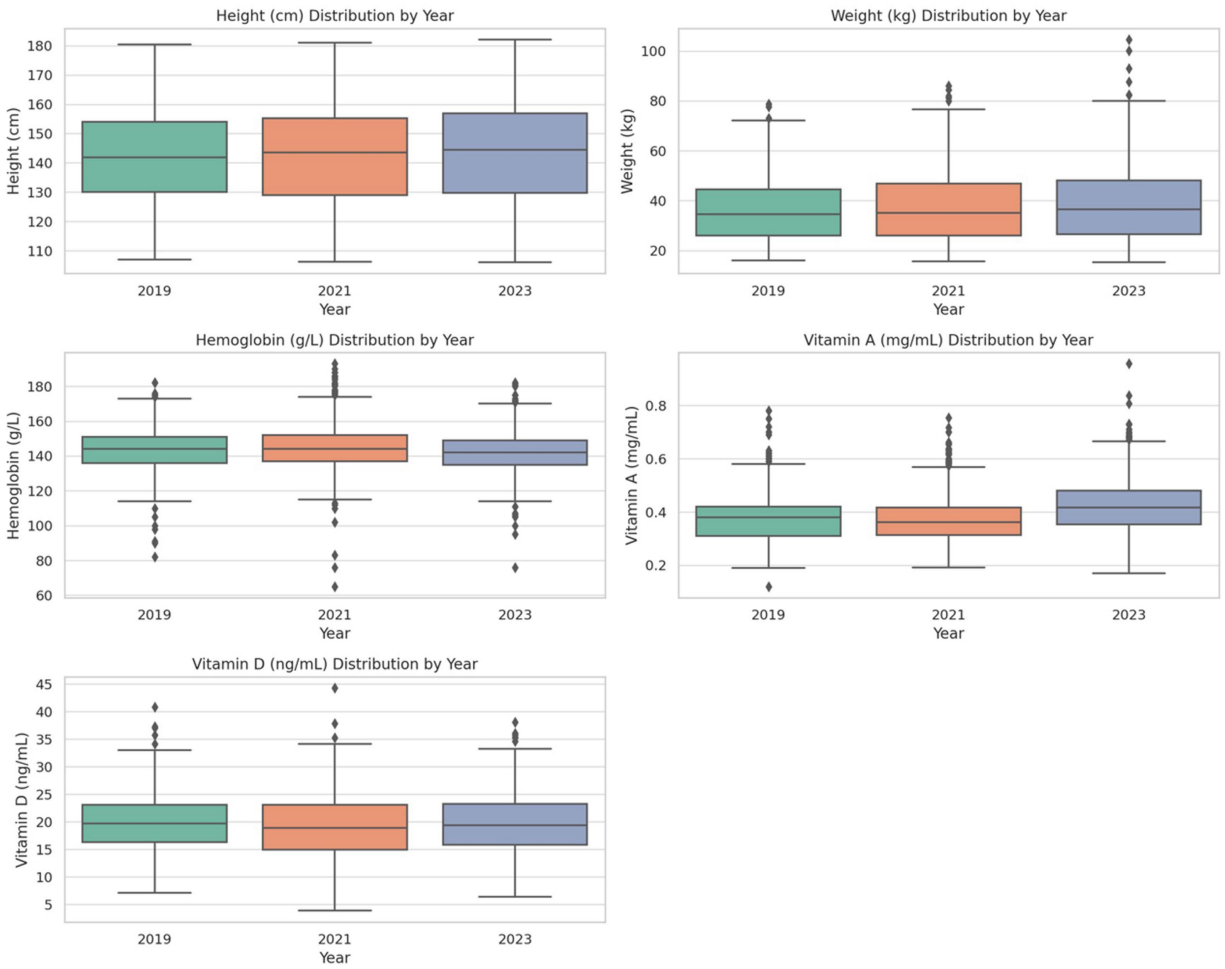
Yearly distributions of nutritional indicators among rural students. Boxplots display the distribution of height (cm), weight (kg), hemoglobin (g/L), Vitamin A (mg/mL), and Vitamin D (ng/mL) for the years 2019, 2021, and 2023. Central boxes represent the interquartile range (IQR), with horizontal lines indicating the median. Whiskers extend to 1.5 × IQR, and outliers are shown as individual dots. Results reveal increasing trends in height, weight, and Vitamin A over time, with moderate variability in hemoglobin and Vitamin D. These patterns reflect the evolving impact of nutritional interventions under the NNIP-RCES.

Boxplots for each nutritional indicator (Figure 2) illustrate the annual distributions from 2019 to 2023. Height and Weight: Median values steadily increased over time, indicating consistent growth among students. Hemoglobin: A slight decline in median levels was observed in 2023 compared to earlier years, suggesting potential challenges in addressing anemia-related factors. Vitamin A: Improved notably in 2023, reflecting enhanced micronutrient supplementation efforts. Vitamin D: Levels remained stable across years with minimal variation.

Figure 2 visually emphasizes the variability in these indicators and underscores the program’s impact on certain key metrics (e.g., height, weight, and Vitamin A) while identifying areas requiring further improvement (e.g., hemoglobin and Vitamin D).

#### 3.3.3 Post-hoc analysis of yearly variations in nutritional status

Following the multivariate analysis of variance (MANOVA), Tukey’s post-hoc test was performed to identify specific pairwise differences among years for the key nutritional indicators. The detailed results, including mean differences, 95% confidence intervals (CIs), and significance levels (*p*-values), are summarized in Table 5.

**Table 5:** Tukey’s post-hoc test results for nutritional indicators.

Variable	Group 1	Group 2	Mean difference	95% CI	*p*-value
Height	2019	2021	0.5370	−1.0598 ~ 2.2057	0.689
	2019	2023	1.7774	0.1618 ~ 3.3929	0.027*
	2021	2023	1.2044	−0.3934 ~ 2.8021	0.181
Weight	2019	2021	1.1828	−0.1962 ~ 2.5618	0.200
	2019	2023	2.6990	1.3346 ~ 4.0634	<0.001**
	2021	2023	1.5162	0.1668 ~ 2.8656	0.023*
Hemoglobin	2019	2021	1.3178	0.0261 ~ 2.6094	0.044*
	2019	2023	−1.4460	−2.7240 ~ −0.1680	0.022*
	2021	2023	−2.7637	−4.0277 ~ −1.4998	<0.001**
Vitamin A	2019	2021	−0.0056	−0.0149 ~ 0.0037	0.339
	2019	2023	0.0447	0.0355 ~ 0.0540	<0.001**
	2021	2023	0.0503	0.0412 ~ 0.0595	<0.001**
Vitamin D	2019	2021	−0.6730	−1.2286 ~ −0.1175	0.013*
	2019	2023	−0.1590	−0.7087 ~ 0.3908	0.776
	2021	2023	0.5141	−0.0296 ~ 1.0577	0.776

The results indicate: Height: A significant difference was observed between 2019 and 2023 (mean difference = 1.7774, *p* = 0.027), while no significant differences were found between 2019 and 2021 (*p* = 0.689) or 2021 and 2023 (*p* = 0.181). Weight: Significant differences were observed between 2019 and 2023 (mean difference = 2.6990, *p* < 0.001) and 2021 and 2023 (mean difference = 1.5162, *p* = 0.023), but not between 2019 and 2021 (*p* = 0.200). Hemoglobin: Significant differences were found between all year pairs (*p* < 0.05), with a consistent declining trend over time. Vitamin A: Significant differences were observed between 2019 and 2023 (mean difference = 0.0447, *p* < 0.001) and between 2021 and 2023 (mean difference = 0.0503, *p* < 0.001), reflecting improvements in Vitamin A levels due to micronutrient supplementation. Vitamin D: A significant difference was noted only between 2019 and 2021 (mean difference = −0.6730, *p* = 0.013), with no significant changes detected in other year pairs.

Overall, significant mean differences were observed for height, weight, hemoglobin, and Vitamin A levels across years, while Vitamin D exhibited relatively stable trends, with significant differences limited to individual year comparisons.

#### 3.3.4 PCA analysis of nutritional indicators over years

**Figure 3:**
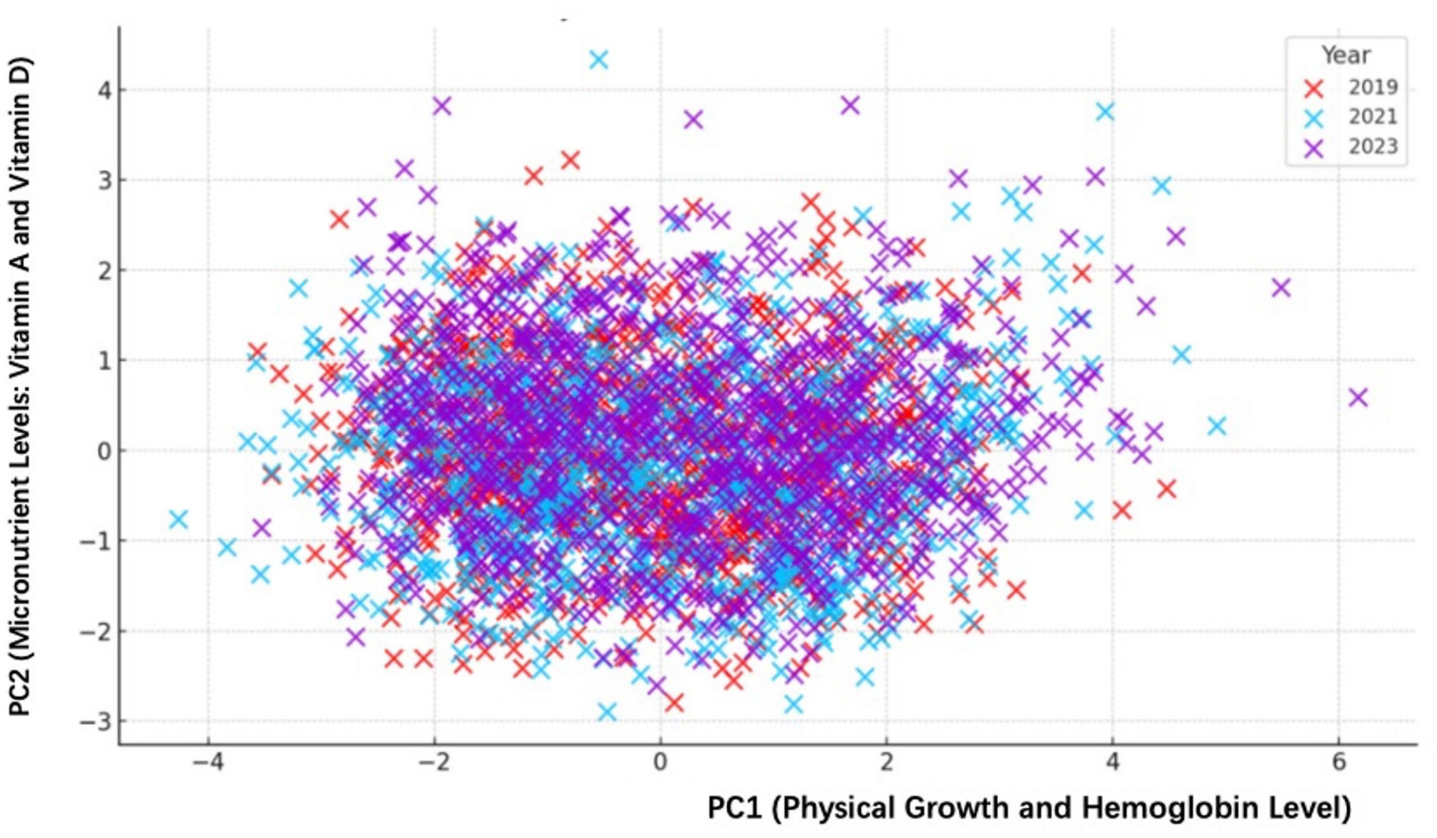
PCA analysis showing changes in nutritional indicators (2019–2023). PCA scatterplot illustrating variations of nutritional status in rural school-aged children from three counties in Yunnan Province over three observation periods (2019, 2021, and 2023). Principal Component 1 (PCI) mainly represents physical growth indicators (height and weight) and hemoglobin levels. Principal component 2 (PC2) primarily captures variations in micronutrients, specifically Vitamin A and Vitamin D levels. Distinct clustering patterns and shifts observed across different years indicate changes potentially linked to nutritional interventions under NNIP-RCES.

Table 6 and Figure 3 illustrate the principal component analysis (PCA) of key nutritional indicators collected in 2019, 2021, and 2023, visually representing changes in the students’ nutritional profiles over these years. The PCA clearly summarizes the data into two main components: the first principal component (PC1) explains about 48.08% of the total variance, while the second principal component (PC2) accounts for 21.47%. Together, these two components capture approximately 69.55% of the overall variability observed.

**Table 6:** PCA loadings for nutritional indicators.

Variable	Principal 1 (PC1)	Principal 2 (PC2)
Height	0.589	−0.104
Weight	0.588	−0.056
Hemoglobin	0.406	0.233
Vitamin A	0.298	0.592
Vitamin D	−0.232	0.762
Eigenvalue (variance)	2.40	48.08
Variance explained (%)	1.07	21.47
Total	3.47	69.55

Figure 3 visually captures how students’ nutritional conditions evolved each year, reflecting the potential effectiveness of the nutritional intervention program. In 2019, depicted by red markers, data points were scattered widely, indicating greater nutritional variability among students at the initial stages of intervention. By 2021, represented by blue markers, the data points began to cluster more tightly, suggesting some progress toward nutritional improvement and greater consistency in the students’ nutritional status. In 2023, marked by purple symbols, the data points clustered even more closely and shifted toward positive values along PC1, clearly highlighting continued improvements, especially related to physical growth (height and weight) and enhanced micronutrient supplementation (notably Vitamin A).

Analyzing each principal component further clarifies their significance. PC1 closely relates to height (factor loading 0.589), weight (0.588), and hemoglobin levels (0.406), thus reflecting overall physical growth and blood oxygen-carrying capacity. On the other hand, PC2 is primarily influenced by Vitamin A (0.592) and Vitamin D (0.762), indicating variations in micronutrient supplementation and nutritional quality.

These PCA findings reinforce the positive outcomes of the nutritional improvement program, showing noticeable progress in both growth and micronutrient levels among students. The movement of data points toward positive values of PC1 over the years clearly illustrates sustained benefits, highlighting that the program’s effects have effectively improved students’ physical development and nutritional health.

## 4 Discussion

This study evaluated the impact of the National Nutrition Improvement Program for Rural Compulsory Education Students (NNIP-RCES) on key anthropometric and nutritional indicators in three counties of Yunnan Province from 2019 to 2023. The findings demonstrate significant progress in physical growth and Vitamin A supplementation but highlight persistent challenges related to anemia prevention and regional disparities.

### 4.1 Nutritional trends and program effectiveness

The results showed significant improvements in key nutrition indicators in 2019, 2021 and 2023, reflecting the positive impact of the student nutrition improvement program. Height and weight gains in all counties have shown that the program is effective in supporting the physical development of school-age children. The significant rise in vitamin A levels further highlights the program’s success in addressing specific micronutrient deficiencies, particularly through targeted interventions.

However, a slight decline in hemoglobin levels has raised concerns about possible problems in the implementation of nutrition improvement programs for rural school-age children. This finding suggests that there is a potential gap between nutrition and health education or iron supplementation and goals or other contributing factors such as inadequate diet or underlying health conditions. Vitamin D plays a key role in bone health and immune function in school-age children (6), and the stable but suboptimal vitamin D levels seen from 3 years of surveillance data also suggest that current interventions may not be sufficient to target this micronutrient supplement.

At the same time, we also need to recognize that school-age children between the ages of 6 and 15 naturally experience an increase in height and weight as part of normal development. Therefore, it is a methodological challenge to distinguish the program-induced improvements from age-related trends. To address this, our analysis utilized a linear mixed-effects model that considered time and region, which allowed us to isolate consistent improvements across counties and years that exceeded the expected biological growth trajectory. Previous studies of NNIP-RCES have reported similar positive changes. For example, Liang et al. (3) observed an increase in average height and weight in rural provinces after the implementation of the program and attributed these changes to the availability of subsidized meals and improvements in dietary intake. Therefore, while the findings suggest that NNIP-RCES has a positive impact, we acknowledge that further quasi-experimental or controlled studies are needed to determine the direct causal relationship.

### 4.2 Regional differences in nutritional status

The results of the implementation of NNIP-RCES vary from county to county. For example, Midu consistently outperformed other counties in terms of height, weight, and vitamin D levels, which may reflect better resource allocation or local implementation strategies. Conversely, Xundian has lower hemoglobin levels, suggesting that the next challenge for the region is to address the region’s anemia problem.

A number of local environmental factors may be associated with the observed decrease in hemoglobin levels. First, rural dietary patterns may have problems with insufficient intake of iron-rich foods such as red meat, poultry, or legumes, leading to persistent iron deficiency. Second, parasitic infections, especially intestinal helminths, are still prevalent in rural Yunnan, exacerbating anemia. In addition, adolescent girls in the study population may have increased nutritional requirements due to menstrual blood loss, which further leads to a decrease in hemoglobin levels. Therefore, we need targeted nutritional and medical interventions in the next step.

### 4.3 Comparison with existing interventions

The results of this study are closely related to global evidence on the effectiveness of school-based nutrition interventions (7). Many similar nutrition intervention programs have been undertaken in other developing countries to study the positive effects of nutritional interventions on physical development (7). However, the reduction in hemoglobin levels differed from other studies (8). Interestingly, vitamin D deficiency also improved after the implementation of the intervention program (9).

The findings from this study are broadly consistent with outcomes reported from similar nutritional intervention programs in neighboring Chinese provinces such as Sichuan and Guizhou. For instance, school nutrition initiatives in Sichuan have successfully improved students’ height, weight, and Vitamin A status, largely through targeted dietary improvements and fortified meal programs (10). In Guizhou, comparable interventions combining nutrition education with meal supplementation have demonstrated positive impacts on physical growth indicators (11). However, the persistent anemia observed in our study contrasts with the more favorable outcomes reported from regions where integrated strategies—including iron supplementation, health education, and regular deworming—have been effectively implemented.

Additionally, comparisons with programs from other low- and middle-income countries (LMICs) reinforce our findings. Countries like India and Kenya have documented significant nutritional improvements when school feeding initiatives are accompanied by comprehensive micronutrient supplementation and robust health education components (12, 13). Conversely, limited improvements in Vitamin D levels observed in our study are consistent with global findings from LMICs, where persistent deficiencies have often been linked to insufficient dietary diversity, seasonal factors, and limited sun exposure, highlighting the need for context-specific solutions.

### 4.4 Suggestions for improving the NNIP-RCES

#### 4.4.1 Enhancing regional equity

To effectively address regional disparities, it is essential to provide focused support and additional resources specifically to rural and economically disadvantaged areas. Rather than using a uniform approach, interventions should be carefully customized based on local dietary practices, resource availability, and community input. This tailored method can genuinely narrow nutritional gaps and support underserved communities more effectively.

#### 4.4.2 Improving micronutrient supplementation

Given persistent micronutrient deficiencies, especially Vitamin D and iron, the program should include fortified foods or targeted supplementation strategies, particularly in regions with limited sun exposure or poor dietary diversity. Emphasizing practical interventions such as incorporating iron-rich local foods, regular anemia screening at schools, and integrating deworming activities can yield significant improvements in children’s health outcomes.

#### 4.4.3 Promoting nutritional health education

Nutrition education should become an engaging and integral part of NNIP-RCES, actively involving students, parents, and communities. Instead of generic advice, educational initiatives should focus on real-life examples and practical skills, such as preparing balanced meals using local ingredients and understanding food safety practices. School curricula should integrate this knowledge effectively, with teachers receiving targeted training to enhance their delivery. Additionally, community workshops should encourage meaningful interactions among families and caregivers, fostering sustainable dietary changes.

#### 4.4.4 Engaging families and communities

Meaningful participation from families and local communities is vital to the success and sustainability of nutrition programs. Stronger partnerships with local governments and agricultural organizations can significantly improve the availability of affordable, nutrient-rich foods. Actively promoting the use of local agricultural products not only supports community food security but also reinforces sustainable agriculture and community development.

#### 4.4.5 Strengthening monitoring and evaluation

Instead of relying solely on periodic checks, ongoing monitoring should incorporate detailed analyses considering gender, age, and specific vulnerabilities. Employing practical and user-friendly feedback mechanisms, alongside effective long-term evaluation approaches, such as community-led reviews and participatory assessments, can better capture real-world impacts and facilitate prompt improvements in program delivery.

### 4.5 Implications for future of NNIP-RCES

#### 4.5.1 Enhancing school infrastructure and meal quality

The construction of school infrastructure has a significant impact on the effectiveness of nutrition interventions. Having adequate kitchen facilities, proper food storage solutions, and clean water sources is essential to ensure that the meals provided in the school are both safe and nutritious. Future research should investigate how changes in school infrastructure affect the quality of meals and the effectiveness of overall program implementation. In addition, funding has been invested in regular practical training of school staff on hygiene, food safety and nutrition standards, as well as in ensuring consistent high-quality meals in different regions.

#### 4.5.2 Addressing socio-economic and environmental factors

Socio-economic and environmental conditions strongly influence the effectiveness of nutrition programs and contribute significantly to regional differences. Schools in more prosperous communities generally benefit from better resources, whereas those in disadvantaged areas frequently encounter logistical and financial challenges. Cultural practices, such as specific dietary preferences and traditional cooking methods, also play an important role in meal acceptance and utilization by students. Future research should closely examine these local factors and develop targeted strategies that effectively meet the unique needs of each community, thus improving nutritional outcomes (14).

#### 4.5.3 Innovative intervention strategies

Integrating emerging digital technologies presents valuable opportunities to enhance the effectiveness and responsiveness of nutritional programs. For instance, practical digital platforms could help monitor meal quality and distribution in real-time, promptly addressing issues as they arise. Additionally, easy-to-use mobile apps can enable straightforward data collection and interactive nutritional education, while also encouraging active participation from students, parents, and community members. Conducting pilot projects to test the usability and effectiveness of these digital solutions in rural settings is crucial to determining their broader applicability and long-term impact.

#### 4.5.4 Long-term effects

Understanding the long-term outcomes of nutrition programs goes beyond measuring immediate health gains. Future research should comprehensively assess how improved nutrition impacts educational achievements, overall physical development, and long-term health, such as reduced chronic disease prevalence. Implementing longitudinal studies that track these effects over time will provide valuable evidence of lasting benefits, helping policymakers and educators design integrated programs that meaningfully connect nutritional support with educational success and overall well-being.

### 4.6 Potential influence of the COVID-19 pandemic

The time span of this study coincides with the global COVID-19 pandemic, which has implications for school operations, public health programs, and child nutrition. In order to maintain data integrity and protect the health of participants, all surveys strictly adhere to local safety precautions. These include pre-screening health screenings, routine temperature monitoring, mandatory mask-wearing, strict hand hygiene, and controlled attendance during medical check-ups and blood collections. In addition, field personnel received professional training, and selected schools were allowed to participate only with permission from the local health authorities. While our study did not provide clarity on the impact of COVID-19 on nutritional status, it is known that some of the changes observed, especially in 2021, may reflect COVID-19 disruptions to household income, dietary quality, and physical activity levels. Although this situation has been mitigated to some extent by the continued implementation of NNIP-RCES, it is critical to identify these external influences when interpreting observed nutritional trends. In future research, we will further integrate the specific impacts of behavioural and socioeconomic dimensions on child nutrition.

## 5 Limitations

This study has several notable limitations. First, while significant nutritional improvements were identified, potential confounding variables may have influenced these findings. For example, this study does not have clear controls for changes in school attendance, disruptions due to local infectious disease outbreaks, or external food assistance programs independent of NNIP-RCES, and may affect nutrition outcomes. Future research should systematically document these external factors to improve the accuracy of causal inference. Second, the stable vitamin D levels observed throughout the study period may be influenced by factors such as seasonal variations in sun exposure, differences in patterns of outdoor physical activity, and relatively limited dietary diversity typical of rural areas. Our current dataset does not include direct measurements of sun exposure or detailed dietary intake data, which limits the extent to which these effects can be quantitatively assessed. These contextual factors will be further investigated in the future to more accurately assess vitamin D status in rural school-age populations.

## 6 Conclusion

NNIP-RCES has significantly improved the nutritional status and physical development of rural school-aged children in China. This study shows clear and consistent improvements in height, weight, and Vitamin A levels, demonstrating the program’s effectiveness in promoting physical growth and reducing specific micronutrient deficiencies. Nevertheless, challenges such as declining hemoglobin levels, persistently inadequate Vitamin D levels, and significant regional disparities remain, highlighting critical areas for further attention and action.

The observed regional differences emphasize the importance of customized strategies and equitable resource allocation, with Midu consistently achieving better outcomes compared to Xundian and Xuanwei. The concerning decline in hemoglobin levels indicates gaps in current anemia prevention measures, highlighting the need for integrated strategies such as iron supplementation, improved dietary diversity, and routine health screenings. Additionally, unchanged Vitamin D levels call for fortified food initiatives or targeted supplementation, particularly in regions with limited dietary variety or sun exposure.

Moving forward, research should focus on examining the program’s long-term impacts on educational achievement, health outcomes, and chronic disease prevention, while also addressing underlying socio-economic and environmental influences. Leveraging digital innovations for real-time monitoring, enhancing community engagement, and strengthening inter-sectoral collaboration is crucial. Policymakers must urgently commit to these improvements to ensure lasting health benefits and educational equity for vulnerable rural children, creating a foundation for lifelong well-being and sustainable community development.

## Data Availability

The data analyzed in this study is subject to the following licenses/restrictions: the data presented in this study are available on request from the corresponding author. The data are not publicly available due to ethical restrictions. Requests to access these datasets should be directed to xufang@kmmu.edu.cn.
